# New insight into the structure and function of Hfq C-terminus

**DOI:** 10.1042/BSR20140128

**Published:** 2015-04-28

**Authors:** Emilie Fortas, Federica Piccirilli, Antoine Malabirade, Valeria Militello, Sylvain Trépout, Sergio Marco, Aziz Taghbalout, Véronique Arluison

**Affiliations:** *Laboratoire Léon Brillouin, CEA–Centre de Saclay, 91191 Gif-sur-Yvette, France; †UMR 12 CEA/CNRS, 91191 Gif-sur-Yvette, France; ‡Department of Physics and Chemistry, University of Palermo, Viale delle Scienze, Ed. 18, 90128 Palermo, Italy; §Institut Curie, Centre de Recherche, Campus Universitaire d'Orsay, bât 112, 91405 Orsay Cedex, France; ║INSERM U1196, Campus Universitaire d'Orsay, bât 112, 91405 Orsay Cedex, France; ¶Université Paris Sud, 91405 Orsay Cedex, France; ¶¶Department of Molecular Biology and Biophysics, University of Connecticut Health Center, 263 Farmington Avenue, Farmington, CT 06032, U.S.A.; **Univ Paris Diderot, Sorbone Paris Cité, 75013 Paris, France

**Keywords:** amyloid fibrils, cellular compartmentalization, post-transcriptional regulation, ribonucleic acid (RNA) processing and degradation, small non-coding ribonucleic acid (RNA), sub-membrane macromolecular assembly, CTR, Hfq C-terminal region, FSD, Fourier self-deconvolution, FTIR, Fourier transform infrared spectroscopy, HfqCTR_p_, Hfq C-terminal peptide, IDP, intrinsically-disordered proteins, ncRNA, regulatory non-coding RNA, NTR, N-terminal region, sRNA, small non-coding RNA, ThT, thioflavin T, WT, wild-type

## Abstract

Accumulating evidence indicates that RNA metabolism components assemble into supramolecular cellular structures to mediate functional compartmentalization within the cytoplasmic membrane of the bacterial cell. This cellular compartmentalization could play important roles in the processes of RNA degradation and maturation. These components include Hfq, the RNA chaperone protein, which is involved in the post-transcriptional control of protein synthesis mainly by the virtue of its interactions with several small regulatory ncRNAs (sRNA). The *Escherichia coli* Hfq is structurally organized into two domains. An N-terminal domain that folds as strongly bent β-sheets within individual protomers to assemble into a typical toroidal hexameric ring. A C-terminal flexible domain that encompasses approximately one-third of the protein seems intrinsically unstructured. RNA-binding function of Hfq mainly lies within its N-terminal core, whereas the function of the flexible domain remains controversial and largely unknown. In the present study, we demonstrate that the Hfq-C-terminal region (CTR) has an intrinsic property to self-assemble into long amyloid-like fibrillar structures *in vitro*. We show that normal localization of Hfq within membrane-associated coiled structures *in vivo* requires this C-terminal domain. This finding establishes for the first time a function for the hitherto puzzling CTR, with a plausible central role in RNA transactions.

## Abstract 2

We showed that Hfq C-terminal region (CTR) has an intrinsic property to self-assemble into amyloid-like fibrils. This region is required for cellular assembly of Hfq into membrane-associated coiled structures. The work establishes a new function for this naturally unstructured Hfq domain.

## INTRODUCTION

A bacterial cell has multiple protein assemblies, which form higher order cellular structures and play important roles during cellular division [1]. We have shown that another family of proteins, those involved in RNA metabolism, also forms cellular assemblies organized into a membrane-associated framework, whose cellular pattern resembles previously described long-range order bacterial cytoskeletal-like structures [2–5]. This cellular framework includes the RNA chaperone Hfq (Host Factor for phage Q beta RNA replication), an abundant and conserved protein found in a variety of bacteria [2,6]. One of the best-characterized functions of Hfq is to modulate post-transcriptionally gene expression via its interaction with various nucleic acids, including regulatory non-coding RNAs (ncRNAs) [6,7]. Currently, approximately 30000 sequences of ncRNA from different species have been identified and among them, those from bacteria are in average 100 nts long, hence their name of small ncRNA (sRNA) [8,9]. The sRNA often functions by base-pairing within regions around the translation initiation signal of associated mRNA target and therefore acts on both mRNA translation and mRNA stability [10]. Annealing of the regulatory sRNA to its cognate mRNA requires Hfq [11,12]. Due to the diversity of mRNA targets of sRNA, Hfq is thus involved in diverse metabolic pathways such as sugar transport, membrane remodelling or quorum sensing [13–15]. Indeed, Hfq is a pleiotropic regulator and nearly a half of Hfq-bound sRNA regulates the expression of membrane proteins or proteins involved in membrane-related cellular processes [16,17]. The cellular compartmentalization of Hfq within the cell periphery could thus play a major role in regulating the expression of these membrane proteins.

The 66 kDa homo-hexameric Hfq protein belongs to Sm-like family of proteins [18]. Sm or Sm-like proteins are commonly made of approximately 100 amino acids that typically fold into a bent five-stranded antiparallel β-sheet capped by an N-terminal α-helix. The individual β-strands of adjacent monomers assemble into a continuous intermolecular antiparallel β-sheet to form a characteristic tore-shaped structure with a charged pore [19]. Though Hfq differs from Sm proteins in a way that Sm proteins fold into heptameric toroid [18], Hfq and Sm proteins have in common the ability to polymerize into well-ordered fibres *in vitro* [20,21].

As for Sm proteins, it has been possible to obtain 3D-structures for Hfq of various bacteria [22–25]. Nevertheless, most of Hfq 3D structures lack the C-terminal region (CTR) and include only the N-terminal region (NTR) of the protein, i.e. the Sm-like domain. This NTR (Sm) region presents two main RNA-binding sites: one on the proximal face (the N-terminal α-helix containing face) with a strong specificity for U-rich RNA and the second RNA-binding site on the opposite distal face with specificity for polyA [26]. Other Hfq regions such as the lateral surface or CTR could also play a role in RNA recognition and binding [27].

The determination of a high-resolution structure for Hfq CTR that makes one-third of the size of *Escherichia coli* Hfq remains elusive [28]. The CTR seems to extend outside the Sm-core and to be intrinsically non-structured [29,30]. This domain has been shown to firm up the subunit interface of the toroidal-shaped N-terminal domain hexamer and is presumably important for the protein stability [29,31]. Hfq-mediated sRNA regulation has been primarily evidenced in bacteria containing Hfq proteins with a CTR extension. However, the role of the CTR in the Hfq-based riboregulation is controversial. Hfq that lack CTR binds some RNA and mediates efficiently the associated riboregulation [31,32]. Conversely, the lack of the CTR could affect other sRNA-mediated regulations [28,29,33,34] and some residues within the CTR have been shown to interact with several RNA species [28,35].

In the present work, we demonstrate that the CTR of Hfq has an unexpected and outstanding property, as it is responsible for the self-assembly of the protein into long fibrils. This feature is correlated to the formation of supramolecular structures by the protein close to the bacterial inner membrane *in vivo*. This finding opens new perspectives on the role of this puzzling C-terminal extension in Hfq-dependent riboregulation.

## EXPERIMENTAL

### *E. coli* strains and plasmids

MC1000 ∆*hfq* strain (AT165) was constructed as described previously in Taghbalout et al. [2]. Full-length and truncated Hfq proteins (Hfq-NTR amino acid residues 1–72 and Hfq-CTR amino acid residues 65–102) were expressed from a plasmid under the control of the arabinose-inducible *P_BAD_* promoter. This plasmid has been previously described in Sledjeski et al. [36]. The Hfq-NTR and Hfq-CTR plasmid constructs derived from this plasmid were made with QuickChange mutagenesis kit (Agilent) by replacing the seventy-third codon with a translation stop signal (for pHfq-NTR) or by deleting the sequence between the second and sixty-fourth codons (for pHfq-CTR). The plasmids were transformed into MC1000 ∆*hfq* strain and the expression of the proteins was induced by using a wide range of arabinose concentrations including conditions that gave normal levels of Hfq full-length expression, as previously shown by quantitative Western blot [37].

### Hfq C-terminal peptide synthesis and preparation

The peptide corresponding to the CTR domain (residues 64–102) is referred as HfqCTR_p_ (Hfq C-terminal peptide) throughout the manuscript. The HfqCTR_p_ (SRPVSHHSNNAGGGTSSNYHHGSSAQNTSAQQDSEETE) was synthetized by Genosphere Biotech (France) using Fmoc chemistry and solid-support resin. After cleavage, the peptide was purified by reverse phase chromatography with an octyl carbon chain (C_8_)-bonded silica column and lyophilized.

HfqCTR_p_ peptide was reconstituted in phosphate buffer 100 mM, pH 7, at 20 mg/ml. This buffer could be either hydrogenated or deuterated for FTIR (Fourier transform infrared spectroscopy) analyses. The self-assembly of HfqCTR_p_ into large fibres is not instantaneous. The time needed to observe aggregation is dependent on the temperature and ranges from few days at 37°C to 2 weeks at 4°C.

### EM analyses of Hfq peptides

#### Sample preparation

A 5 μl drop of HfqCTR_p_ sample at 1 mg/ml in phosphate buffer 50 mM, pH7, (i.e., stock solution diluted 1:20 in water) was deposited on a glow discharged carbon-coated EM copper grid (200 mesh square grid, EMS). After 5 min, the excess of sample was blotted out using a Whatman filter paper and then the grid was washed with water three times to eliminate phosphate buffer, which would react with uranyl acetate used for negative staining. To perform negative staining, 5 μl of uranyl acetate solution (2%) was applied on to the grid containing peptidic sample. After 30-s incubation, the excess of uranyl acetate was blotted out and then the grids were kept in a dry, dark, dust-free environment until observation with the electron microscope.

#### Sample observation

The EM grid was then mounted on to a room temperature-equilibrated holder and subsequently introduced into a JEOL 2200FS electron microscope (JEOL). Images (2048×2048 pixels) were acquired using a ssCCD camera (Gatan UltraScan 894) at 40000× (nominal magnification, the corresponding pixel size was 0.32 nm).

### FTIR spectroscopy analysis of HfqCTR_p_

Infrared absorption measurements have been performed in the region between 4000 and 400 cm^−1^ through the use of a Vertex70 interferometer coupled with a conventional IR source and a Triglycine sulfate (DTGS) detector. The HfqCTR_p_ fibrils sample (20 mg/ml) has been loaded into a conventional liquid cell equipped with CaF_2_ windows and a Teflon spacer 6-μm thick. A total of 256 scans were collected and five independent measurements were averaged. Spectra were baseline corrected and background subtracted before analysis. Qualitative evaluation of secondary structure has been performed through the combination of second derivative and Fourier self-deconvolution (FSD) analyses [38]. Overlapping peaks composing the amide I band were fitted with Voigt profiles with a bandwidth varying between 15 and 25 cm^−1^.

### Confocal fluorescence microscopy analysis of HfqCTR_p_

HfqCTR_p_ sample was diluted 1:100 and stained with thioflavin T (ThT), a benzothiazole salt which it is widely used to visualize and quantify the presence of amyloid-like aggregates, both *in vitro* and *in vivo* [39]. Ten microlitre aliquots of stained HfqCTR_p_ samples were placed on microscope slides and imaged at 1024×1024 pixel resolution using a Leica RCS SP5 confocal laser scanning microscope with a 63× oil objective numerical aperture (NA)=1.4 (Leica Microsystems), at a scanning frequency of 400 Hz. The laser excitation was set at 415 nm and emission detected in the range 450–500 nm.

### *In vivo* immunofluorescence microscopy

Immunofluorescence (IF) experiments were done as described previously [2,40]. Briefly, wild-type (WT) MC1000 [41], MC1000 *∆hfq/P_ara_-hfq* or MC1000 *∆hfq/P_ara_-hfq^1–72^* cells were fixed in the growth medium for 30 min at room temperature in the presence of 2% formaldehyde and 0.016% glutaraldehyde and then adsorbed on silane-coated cover slips. Cells were stained with purified rat anti-Hfq antibody [2]. Rat antiserum directed against untagged-Hfq (Eurogentech) was purified by absorption to purified Hfq bound to a PVDF membrane followed by an elution with 0.2 M glycine (pH 2) and a renaturation with 1.5 M Tris-base (pH 8.8) [2]. Alexa fluor 488-conjugated goat anti-rat secondary antibodies (Molecular Probes) were used to detect the stained Hfq cellular proteins. Images were collected using the Volocity image acquisition program (Perkin–Elmer). Images were not subjected to deconvolution and were captured at 520–550 nm using the filter set # 89006 (Chroma Technology). Cells (250–300) were analysed for each condition and the described localization patterns were present in most of the cells. Image processing was done using Volocity (Perkin–Elmer) and Photoshop (Adobe) softwares.

## RESULTS

### HfqCTR_p_ folding and self-assembly

In order to probe for the presence of secondary structure within the synthesized CTR Hfq peptide, we subjected the peptide to CD and FTIR analyses. We recorded the spectra few hours after dissolving lyophilized HfqCTR_p_ in phosphate buffer. None of the spectra indicated the presence of secondary structure (result not shown). This result was in agreement with the high frequency of amino acids-promoting disorder (such as A, N, Q, E, G, K, H, D and S) and with an intrinsically-disordered protein (IDP) [42,43].

**Figure 1:**
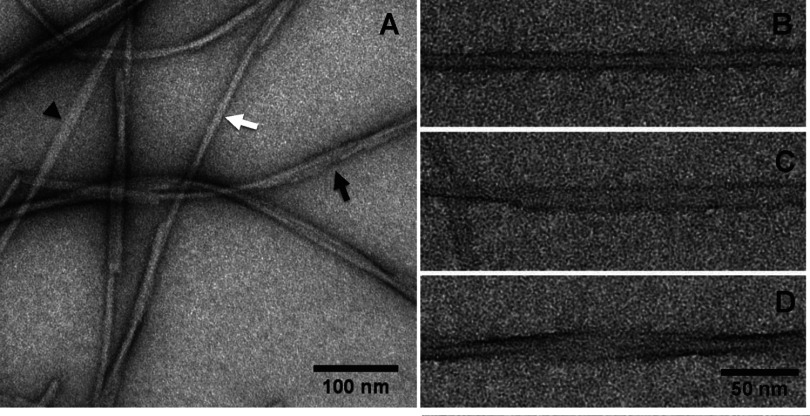
TEM negative staining micrographs showing Hfq C-terminal fibres (**A**) General microscopy field of Hfq C-terminal sample, illustrating the heterogeneity in fibres organization: closed (white arrow), opened (arrowhead) and twisted (black arrow) fibres. (**B**–**D**) Close-up views of closed, opened and twisted fibres respectively. Scale bars are 100 nm (**A**) and 50 nm (**B**–**D**).

After few days (the time being dependent on the temperature, see ‘Experimental’), the HfqCTR_p_ peptide solution undergoes changes. The solution became very viscous and presented small particles. To understand the nature of these changes, we subjected the peptide solution to negative staining TEM analysis. This revealed the presence of long fibrillar structures (Figure 1), indicating a tendency of C-terminal Hfq peptide to self-assemble into long-ordered structures. Similar preparation with the HfqNTR_p_ did not form any long-ordered structures (Supplementary Figure S1). HfqCTR_p_ fibrils revealed by negative staining EM seem to be monodisperse in width but with varying lengths (up to several micrometres). The width of the fibrillar structures as measured perpendicularly to the longitudinal axis is approximately 10 nm. TEM also evidences that these fibrils are twisted and sometimes entangled to form larger bundles of fibrils (Figure 1).

**Figure 2:**
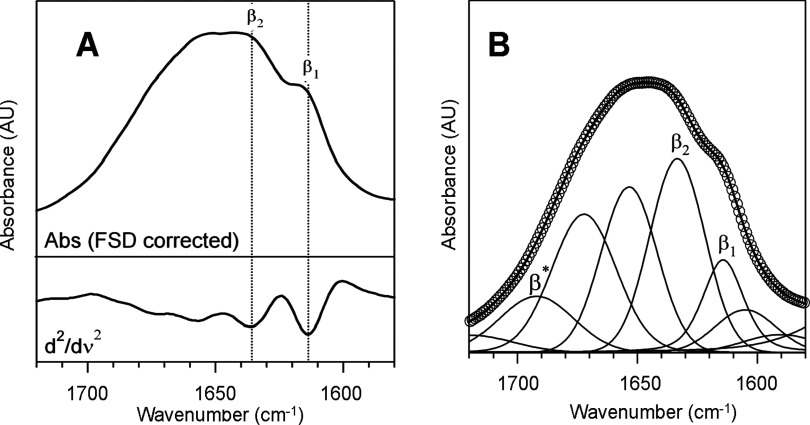
Secondary structure of fibrillar Hfq CTR from FTIR absorption spectroscopy (**A**) Amide I FSD corrected spectrum (top) and second-derivative spectrum (down) of C-terminal Hfq fibrils. Two β-sheet moieties, β_1_ and β_2_, are clearly detected. (**B**) Experimental (circles) and fitted (lines) data of amide I band of C-terminal Hfq fibrils. Two distinct β-sheet structures are present in the aggregates, β_1_ and β_2_ and the presence of the high frequency component β* suggests the possible presence of antiparallel moieties [51].

Proteins could show a high tendency to form fibrillar aggregates under certain thermodynamic conditions. The aggregates referred to as amyloid fibrils, reported for a substantial number of proteins, are elongated structures whose spine is formed by highly ordered β-sheets running parallel to the fibril axis [44]. Amyloid aggregation is considered as a general characteristic of proteins, being the amyloid fibril the most stable state for the polypeptide chain [45]. In particular, there are several examples of proteins forming amyloid fibrils whose native state lacks of a well-defined secondary structure or belongs rather to the IDP family [46]. The observed self-assembly into fibrillar extended structures of HfqCTR_p_ was thus quite interesting given the fact that secondary structures was absent from freshly dissolved peptide. We probed thus for the presence of amyloid-like secondary structures in Hfq peptide after self-assembly by using FTIR spectroscopy. The sensitivity of amide vibrations to secondary structures makes FTIR an excellent probe for protein ordering, especially for β-sheet like structures [47]. As shown in Figure 2, FTIR absorption spectrum of HfqCTR_p_ fibrils in the region of amide I (1720–1560 cm^−1^) indicates the band is peaked approximately 1648 cm^−1^ with a remarkable shoulder at approximately 1615 cm^−1^, suggesting that peptides in the aggregates are arranged into at least two secondary structural moieties [48,49]. The second derivative of the curve, together with FSD-enhancement technique allowed identifying the different spectral components of amide I band (Figure 2A), which were used for initializing the curve fitting procedure (Figure 2B). Five main moieties was detected, absorbing at approximately 1616 and 1634 cm^−1^, both of them corresponding to vibrational motions of the backbone amide moieties in β-sheet conformations, 1655 cm^−1^, ascribed to random coil peptidic segments, 1673 cm^−1^, typical amide I frequency of turns and 1690 cm^−1^ which corresponds to the amide I high frequency component of β-sheet vibrations, due to excitonic splitting, in case of structures arranged in an antiparallel fashion [50,51]. The results obtained from fitting procedure revealed that approximately 42% of the total amide I area (18% ascribed to the peak at approximately 1616 cm^−1^ and 24% due to the one approximately 1634 cm^−1^) is arranged in β-sheet conformation whereas only 21% of the peptide shows a random coil arrangement. The observation of a high content of β-sheets reflects the aggregated nature of the sample. In particular, the low frequency β-sheet moiety, at approximately 1616 cm^−1^, can be associated to multi-stranded intermolecular β-sheet and resembles the typical fingerprint of the cross-β motif found in amyloid-like fibrils [52,53].

**Figure 3:**
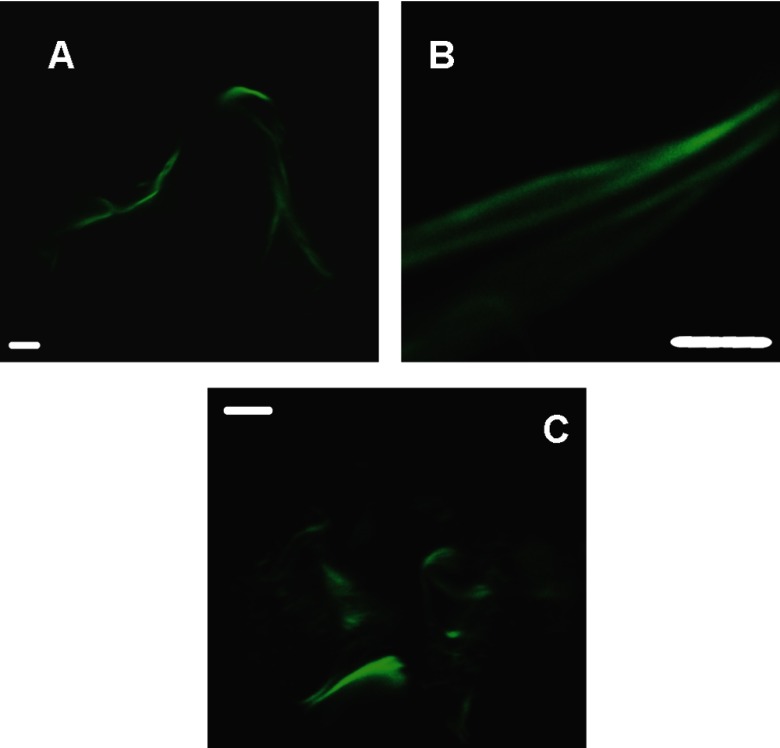
Confocal microscopy fluorescence images for CTR Hfq fibrils Samples (Hfq-CTR peptide) were stained by ThT as indicated under ‘Experimental’ section. (Scale bar=5 mm). Distinct morphologies are present in the sample: laterally packed fibrils bundles (**A** and **B**) or clustered wires (**C**). Scale bar=5 μm.

The evidence of a substantial β-sheet structure together with the fibrillar morphology of HfqCTR_p_ aggregates suggests an assembly via a similar mechanism that lead to formation of amyloids. To test whether the observed fibrillar structures are similar to amyloids, we used ThT stain that is commonly used to detect amyloid structures (see ‘Experimental’ for details). Stained fibrils were imaged through confocal fluorescence microscopy [54]. As seen on Figure 3, the enhanced fluorescence of ThT confirms the presence of intermolecular β-sheet distributed along the fibrils. They furthermore highlight two distinct morphologies of the aggregates present in the sample: laterally packed fibrils bundles (Figures 3A and 3B) as well as clustered wires with diameters smaller than 1 μm (Figure 3C).

### Hfq-CTR is required for assembly of the Hfq high-order cellular structures

**Figure 4:**
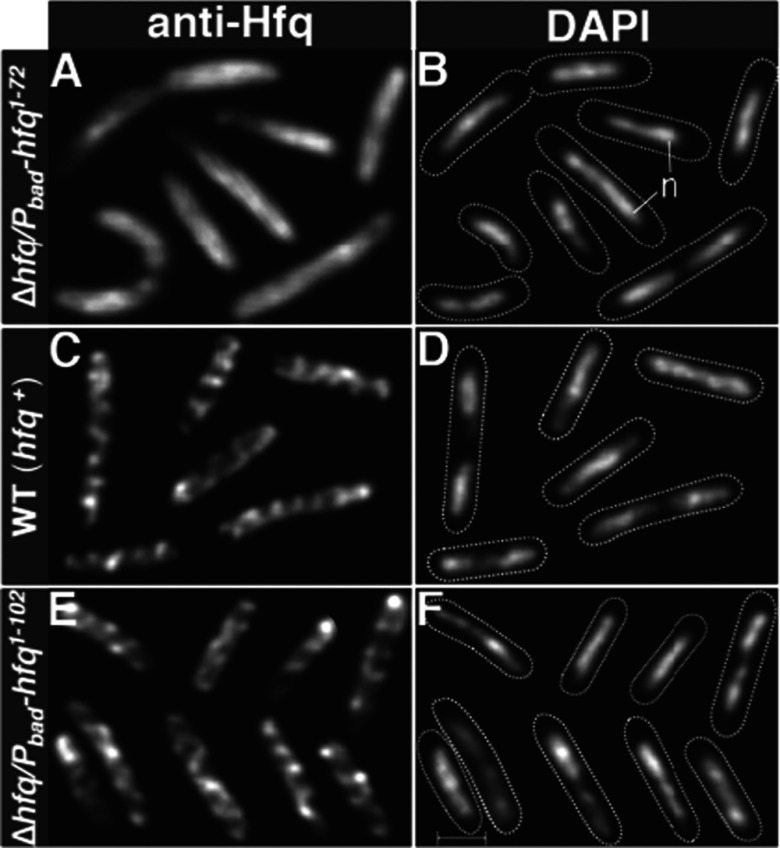
Assembly of Hfq higher-order cellular structures requires the C-terminal domain of Hfq (**A**, **B**, **E** and **F**) *hfq* Null cells expressed Hfq under the control of *P_ara_* promoter plasmid-encoded truncated Hfq**^(1–72)^** (**A** and **B**) or plasmid-encoded full-length Hfq (**E** and **F**). (**C** and **D**) WT cells expressed chromosomally encoded WT Hfq (**C** and **D**). Cells were doubly stained with DAPI, for nucleoid staining and by IF using a purified anti-Hfq polyclonal antibody. Cells shown in the composite micrographs represent cells selected from different areas of one field and are representative of most cells in the indicated experiments. In DAPI micrographs dotted lines show boundaries of cells and n indicates nucleoid. Scale bar=1 μm.

We previously reported and illustrated in the present study IF microscopy using WT cells (Figure 4C). Cells stained with a purified anti-Hfq antibody showed that Hfq, unlike cytoplasmic proteins, is not diffusely distributed within the cell, but is localized as repetitive extended elements oriented roughly at 45° angles along the axis of the cell (Figure 4C) [2]. The structures appear to coil around the cell periphery suggesting a membrane association of the Hfq cellular fibril-like structures. To determine the role of the self-assembly property of the C-terminal domain in the formation of such higher order cellular structures observed with full-length Hfq in intact WT cells, we carried IF localization studies in cells that lacked Hfq CTR. The cells expressed under the control of arabinose promoter a plasmid encoded full-length Hfq or Hfq protein without the C-terminal domain (HFq-NTR, amino acid residues 1–72) or the C-terminal domain of the protein alone (HFq-CTR, amino acid residues 65–102). Because the limit between NTR and CTR is not formally known, seven overlapping residues (residues 65–72) were included in both NTR and CTR constructs. This showed that the HFq-NTR domain, which lacks the C-terminal extension of the full-length Hfq, was uniformly distributed throughout the cytoplasm without any detectable organization (Figure 4A). In contrast, full-length Hfq expressed from the plasmid showed similar organized cellular structures observed in WT cells expressing chromosomally-encoded Hfq (Figures 4C and 4E). These localization patterns were observed over a wide range of inducer concentrations (including conditions that give normal levels of expression of Hfq full-length [37]), indicating that the observed diffuse localization is not due to under or overexpression of the protein. Taken together, the intrinsic property of the CTR to self-assemble with the fact that loss of CTR domain of Hfq was associated with loss of assembly of higher order cellular structures observed with full-length proteins, strongly suggest a direct role of the CTR domain of Hfq in the macromolecular assembly of Hfq cellular structure in the vicinity of the membrane of *E. coli* bacterium.

Attempts to test the ability of the CTR domain to assemble within long-range cellular structures in the absence of the rest of the protein were not successful due to inability to stably express the CTR domain. The CTR domain expressed alone, apart from NTR, is presumably very unstable and degraded quickly *in vivo* as no corresponding band was detected in Western blot analyses of extract from cells grown in the presence of a range of arabinose concentration (result not shown). This is consistent with protein instability prediction analysis for this region [55].

## DISCUSSION

Collectively, our results evidence that Hfq CTR has a strong propensity to self-associate and to form amyloid-like fibrillar structures. The assembly of the Hfq-CTR fibrillar structures was shown by negative staining EM, thioflavin staining fluorescence microscopy and FTIR spectroscopy. The self-assembly of the full-length Hfq protein in amyloid-type fibres has been previously reported [21]. Nevertheless, the region of the protein required for the self-assembly was not clearly identified. In the present study, we show that Hfq-CTR has an intrinsic property to self-assemble independently of the rest of the protein, whereas the Hfq-NTR fails to assemble into extended structures. The present work reveals that Hfq-CTR is required for the self-assembly of Hfq into long-range order cellular structures. Furthermore, as fibrillar Hfq polymers retains their RNA-binding properties [21], this work shed light on how Hfq CTR region could have a physiological function in directing Hfq cellular localization in order to govern its activity. Note that previous reports showed that the NTR (core Sm-like domain) alone retains most Hfq RNA-binding properties [31,32], indicating that the loss of cellular localization of Hfq that lacks the CTR is not due to failure in RNA binding.

Previous studies indicated that this Hfq CTR fragment is intrinsically disordered [30] and that IDPs often have a tendency to form amyloid-like structures [56]. Formation of amyloid-type fibrillar structure *in vitro* shows that Hfq-CTR domain belongs to this family of amyloid IDPs prone to aggregation. We show in the present study that self-assembly of the CTR region is associated with formation of different secondary structures within the CTR fragment as indicated by the changes in FTIR spectra observed after assembly of the fibrillar structures. The mechanism underlying folding and assembly of such a natively unstructured domain of Hfq remains to be elucidated. Whether interactions with other cellular components such as RNA molecules could play a role in accelerating the observed slow folding/self-assembly process is not known. Flexible IDP regions have, for instance, been shown to favour interactions with large cellular partners, like nucleic acids and have been found in some RNA chaperones [57]. This could apply to Hfq whose CTR could play an additional role in RNA recognition [28].

Amyloid fibrils are usually the hallmark of neurodegenerative diseases, but they have also been reported in bacteria, in particular for proteins that form curli at the bacterial cell surface [58,59]. Commonly, intracellular amyloid-type protein aggregation is considered potentially toxic for bacteria [60]. Nevertheless, it has been shown that some proteins can gain new functions when present as amyloid fibrils. For example, the hydrogenase maturation factor HypF N-terminal domain in its aggregated form binds cell membrane and DNA whereas its monomeric form does not [61]. In the present study, we present a new example where a bacterial amyloid-like self-assembly plays an important role in directing assembly of Hfq into supramolecular cellular structures that includes other components of the RNA processing and degradation pathway. How self-assembly of the CTR tail of Hfq might lead to the formation of the organized cellular structures seen in intact cells remains to be elucidated. Understanding of temporal and spatial self-assembly process of Hfq will thus be paramount to understand how post-transcriptional regulation occurs as a function of the cell cycle.

## Online data

Supplementary data
