# Supplementary material for: New insight into the structure and function of Hfq C-terminus
Source: Biosci Rep. 2015 Apr 28;35(2):e00190. doi: 10.1042/BSR20140128 (PMC4413018; doi:10.1042/BSR20140128)
Supplement: Supplementary data [file bsr035e190ntsadd.pdf]

Figure S1 Comparison of HfqCTRp (left) and HfqNTRp (right) self-assembly. TEM negative staining micrographs of HfqCTRp and HfqNTRp. Peptides were reconstituted in phosphate buffer 100 mM pH7 at 20 mg/mL for few days and then diluted for TEM analysis (see Experimental). Only HfqCTRp showed self-assembly into extended fibrillar structures whereas the HfqNTRp did not. The peptide, HfqNTRp, corresponding to Hfq N-terminal region (residues 1 to 72) was synthesized based on the following sequence:
MAKGQSLQDPFLNALRRERVPVSIYLVNGIKLQGQIESFDQFVILLKNTVSQMVYKHAISTVVPSRPVSHHS

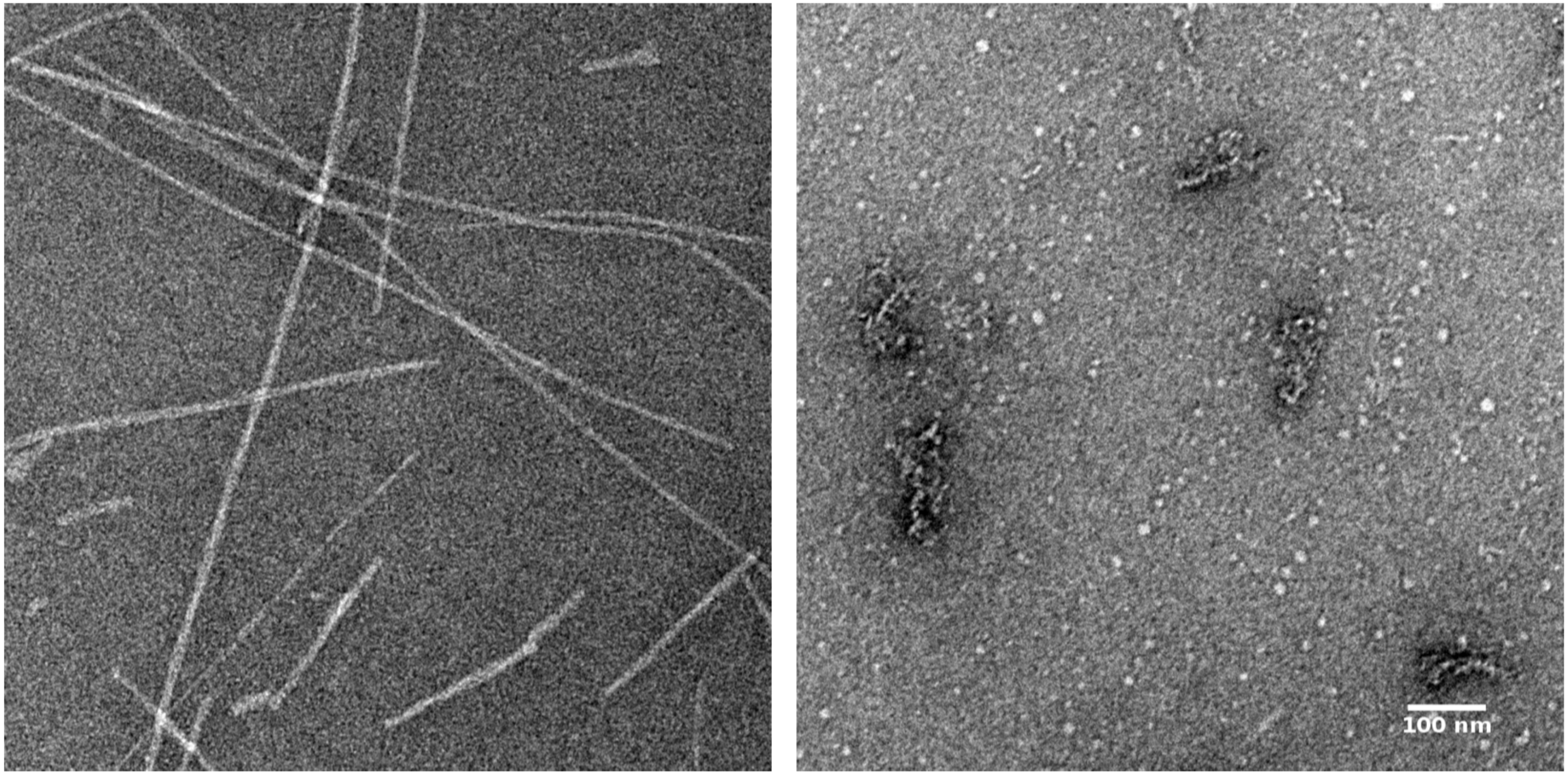

Figure S1
